# Inhaled amikacin for pneumonia treatment and dissemination prevention: an experimental model of severe monolateral *Pseudomonas aeruginosa* pneumonia

**DOI:** 10.1186/s13054-023-04331-x

**Published:** 2023-02-14

**Authors:** Ana Motos, Hua Yang, Gianluigi Li Bassi, Minlan Yang, Andrea Meli, Denise Battaglini, Roberto Cabrera, Joaquim Bobi, Francesco Pagliara, Gerard Frigola, Marta Camprubí-Rimblas, Laia Fernández-Barat, Montserrat Rigol, Antoni Ferrer-Segarra, Kasra Kiarostami, Daniel Martinez, David P. Nicolau, Antonio Artigas, Paolo Pelosi, Jordi Vila, Antoni Torres

**Affiliations:** 1 Servei de Pneumologia i Al•lèrgia Respiratòria, Pneumology Department, Hospital Clínic, Thorax Institute, Calle Villarroel 170, Esc 6/8 Planta 2, 08036 Barcelona, Spain; 2 Institut d’Investigacions Biomèdiques August Pi i Sunyer (IDIBAPS), Barcelona, Spain; 3 University of Barcelona, Barcelona, Spain; 4 Centro de Investigación Biomedica En Red- Enfermedades Respiratorias (CIBERES), Barcelona, Spain; 5 Fondazione IRCCS Cà Granda Ospedale Maggiore Policlinico Internal Medicine Department, Respiratory Unit and Adult Cystic Fibrosis Center, and Department of Pathophysiology and Transplantation, Università degli Studi di Milano, Milan, Italy; 6 Anesthesia and Critical Care, San Martino Policlinico Hospital, IRCCS for Oncology and Neurosciences, Genoa, Italy; 7 Department of Pathology, Hospital Clinic, Barcelona, Spain; 8 Critical Care Center, ParcTaulí Hospital Universitari, Institut d’Investigació i Innovació Parc Taulí (I3PT), Universitat Autònoma de Barcelona, Sabadell, Spain; 9 Anestesiologia i Reanimació, Hospital del Mar - Parc de Salut Mar, Barcelona, Spain; 10 Center for Anti-Infective Research and Development, Hartford Hospital, Hartford, CT USA; 11 Department of Surgical Sciences and Integrated Diagnostics (DISC), University of Genoa, Genoa, Italy; 12 Barcelona Centre for International Health Research (CRESIB), ISGlobal, Barcelona, Spain; 13 Department of Clinical Microbiology, Centre for Biomedical Diagnosis, Hospital Clínic, Barcelona, Spain; 14 Critical Care Research Group, The Prince Charles Hospital, University of Queensland, Queensland University of Technology, UnitingCare Hospitals, Wesley Medical Research, Brisbane, Australia; 15 CIBER de Enfermedades Infecciosas (CIBERINFEC), Instituto Salud Carlos III, Madrid, Spain

**Keywords:** Inhaled amikacin, Severe pneumonia, *Pseudomonas aeruginosa*, Animal model, Multidrug resistance, Monolateral pneumonia

## Abstract

**Background:**

*Pseudomonas aerugino*sa pneumonia is commonly treated with systemic antibiotics to ensure adequate treatment of multidrug resistant (MDR) bacteria. However, intravenous (IV) antibiotics often achieve suboptimal pulmonary concentrations. We therefore aimed to evaluate the effect of inhaled amikacin (AMK) plus IV meropenem (MEM) on bactericidal efficacy in a swine model of monolateral MDR *P. aeruginosa* pneumonia.

**Methods:**

We ventilated 18 pigs with monolateral MDR *P. aeruginosa* pneumonia for up to 102 h. At 24 h after the bacterial challenge, the animals were randomized to receive 72 h of treatment with either inhaled saline (control), IV MEM only, or IV-MEM plus inhaled AMK (MEM + AMK). We dosed IV MEM at 25 mg/kg every 8 h and inhaled AMK at 400 mg every 12 h. The primary outcomes were the *P. aeruginosa* burden and histopathological injury in lung tissue. Secondary outcomes included the *P. aeruginosa* burden in tracheal secretions and bronchoalveolar lavage fluid, the development of antibiotic resistance, the antibiotic distribution, and the levels of inflammatory markers.

**Results:**

The median (25–75th percentile) *P. aeruginosa* lung burden for animals in the control, MEM only, and MEM + AMK groups was 2.91 (1.75–5.69), 0.72 (0.12–3.35), and 0.90 (0–4.55) log_10_ CFU/g (*p* = 0.009). Inhaled therapy had no effect on preventing dissemination compared to systemic monotherapy, but it did have significantly higher bactericidal efficacy in tracheal secretions only. Remarkably, the minimum inhibitory concentration of MEM increased to > 32 mg/L after 72-h exposure to monotherapy in 83% of animals, while the addition of AMK prevented this increase (*p* = 0.037). Adjunctive therapy also slightly affected interleukin-1β downregulation. Despite finding high AMK concentrations in pulmonary samples, we found no paired differences in the epithelial lining fluid concentration between infected and non-infected lungs. Finally, a non-significant trend was observed for higher amikacin penetration in low-affected lung areas.

**Conclusions:**

In a swine model of monolateral MDR *P. aeruginosa* pneumonia, resistant to the inhaled AMK and susceptible to the IV antibiotic, the use of AMK as an adjuvant treatment offered no benefits for either the colonization of pulmonary tissue or the prevention of pathogen dissemination. However, inhaled AMK improved bacterial eradication in the proximal airways and hindered antibiotic resistance.

**Supplementary Information:**

The online version contains supplementary material available at 10.1186/s13054-023-04331-x.

## Background

Current guidelines for the management of hospital-acquired pneumonia (HAP) and ventilator-associated pneumonia (VAP) stress the need for preventive strategies and accurate etiologic diagnosis [1, 2]. Unfortunately, routinely employed antibiotic agents are often ineffective against gram-negative pathogens, with multidrug resistance (MDR) a common problem, even in combination therapy [3]. The treatment of severe gram-negative pneumonia therefore remains a major challenge [3, 4].

*Pseudomonas aeruginosa* pneumonia is usually treated with a combination of intravenous antibiotics to ensure the adequate treatment of MDR isolates [1, 2]. However, insufficient lung distribution and adverse side effects limit this approach [5], leading to inhaled antibiotics gaining increasing attention as a site-specific treatment [6]. Inhaled has have improved bactericidal properties by ensuring high concentrations in tracheal secretions and epithelial lining fluid (ELF) [7, 8], while lowering systemic concentrations to curtail its nephrotoxic and ototoxic effects [9, 10]. Experimental and clinical studies have elicited conflicting results when assessing inhaled AMK in VAP [7, 11–19]. Unfortunately, the latest two randomized clinical trials of inhaled AMK have failed to demonstrate any benefit in primary outcomes (i.e., change in clinical pulmonary infection score and survival at days 28–32, respectively) for mechanically ventilated patients with pneumonia [14, 19]. The most recent management guidelines for HAP/VAP advise against the routine use of adjunctive inhaled therapy, recommending it only for susceptible MDR bacteria [2, 20]. Given that study design, dosing, and nebulization technique may have obscured the impact of inhaled AMK on VAP dissemination and the emergence of resistance [21, 22] further research is warranted.

In this study, we aimed to analyze the effects of inhaled AMK of monolateral pneumonia caused by *P. aeruginosa* in pigs.

## Materials and methods

### Study design

We analyzed the effects of inhaled AMK in a translational large animal (porcine) model of monolateral pneumonia caused by *P. aeruginosa*, according to the ARRIVE guidelines [23]. This study was conducted according to the European guidelines for the Care of Animal Experimentation at the Division of Animal Experimentation, Department of Pulmonary and Critical Care Medicine, Hospital Clínic, Barcelona, Spain. Documented approval from the appropriate Institutional Review Board was obtained prior to the study started (number approval 9772).

### The animal model

Eighteen female Large White-Landrace pigs underwent 96 h of mechanical ventilation. Animal preparation, including anesthesia induction and maintenance, airway management, and hemodynamic invasive monitoring were performed as previously described [24]. Immediately after preparation and stabilization, each animal was challenged with 15 mL of 10^7^ CFU/mL log-phase culture of AMK-resistant and MEM-susceptible *P. aeruginosa* at minimum inhibitory concentrations (MICs) of > 256 and 0.25 mg/L, respectively. We did select *P. aeruginosa* strain resistant to AMK to consider the worst scenario for AMK efficacy, but susceptibility to IV antibiotic was still ensured.

The challenge was instilled bronchoscopically into the right upper, middle, and lower lobes, with the animals kept in a lateral-right slight Trendelenburg position to ensure a right predominance of infection. This was maintained for 24 h, before the lateral position was changed from one side to the other every 6 h. The pneumonia diagnosis was confirmed 24 h after the bacterial challenge based on a decrease in the arterial partial pressure of oxygen (PaO_2_)/fraction of oxygen in the inhaled gas (FiO_2_), plus at least one of the following signs of infection: temperature ≥ 38.5 °C, leukocytosis ≥ 20.10^9^ cells/L, or purulent secretions.

Protocol.

The 18 animals were randomized into one of three groups after creating the model scenario: a control group (CONTROL, *n* = 6), a meropenem group (MEM, *n* = 6), and an amikacin plus meropenem group (MEM + AMK, *n* = 6). The CONTROL group received a 4-mL inhaled solution of 0.9% NaCl every 12 h and no intravenous antibiotics. The MEM group received 25 mg/kg of MEM intravenously every 8 h, plus the inhaled saline. The MEM + AMK group received the inhaled saline and IV MEM, plus a 3.2 mL dose of BAY 41–6551 inhalation solution (125 mg/mL of AMK, 400 mg total) every 12 h. The aerosolized antibiotic was delivered with the recently patented NEKTAR Pulmonary Drug Delivery System (Novartis Pharmaceuticals, San Carlos, CA, USA), which is a high-efficiency vibrating mesh synchronized nebulizer for the intrapulmonary delivery of inhaled AMK [17]. The nebulizer unit was connected to the ventilator circuit between the Y-piece and endotracheal tube. Two cables connected the control module to both the nebulizer and the air pressure-feedback unit (for breath synchronization) in the inspiratory limb of the ventilator circuit [17].

During the procedure, animals were kept prone to favor the bilateral distribution of the aerosol. We maintained the ventilatory settings from before to after nebulization, including positive end-expiratory pressure, tidal volume, respiratory rate, FiO_2_, and humidification (this system is not significantly affected by active humidification [17]). Respiratory, hemodynamic, clinical, microbiological, and inflammatory assessments were recorded at scheduled time points throughout the experiment, as shown in Additional file 1: Figure S1. Drug distribution was analyzed in the MEM and MEM + AMK groups at the first aerosolization or the first meropenem administration, as previously described [15].

The animals were killed 96 h after the bacterial challenge, and the lungs were exposed, excised, and weighed. We obtained three samples from the most affected region of each of the three right and two left lobes for histological, pharmacological, and microbiological assessments. Lung biopsies were processed and analyzed according to previously established protocols [24]. Further details are reported in the Online Data Supplement.

### Statistical analysis

Data are reported as means ± standard deviation or medians (interquartile range) for normally and non-normally distributed continuous variables, respectively. Categorical values are presented as percentages. Differences among study groups and/or assessment times for continuous variables were analyzed by restricted maximum likelihood analysis, based on a repeated-measures approach (including study treatment and times of assessments or lobes). All two-sided comparisons among groups were performed with Bonferroni correction. Differences between categorical variables were analyzed by Fisher’s exact test. A two-sided *p*-value ≤ 0.05 was considered statistically significant. We used SAS 9.4 (SAS Institute Inc., Cary, NC, USA) and GraphPad Prism version 8.0 (GraphPad Software, San Diego, CA, USA) for the statistical analyses.

## Results

### Study populations

Eighteen Large White-Landrace female pigs (32.4 ± 1.8 kg) were included, of which 17 completed the study. One pig in the control group was euthanized at 76 h due to severe respiratory and hemodynamic instability and received only four saline doses by nebulizer.

### AMK nebulization

After meeting the diagnostic criteria for pneumonia (Additional file 1: Table S1), each animal received 7 nebulized doses of either saline (CONTROL and MEM groups) or AMK. Table 1 shows the ventilatory settings during nebulization and the adverse events. The CONTROL group had the highest positive end-expiratory pressure, while inspiratory flow and respiratory rate did not differ significantly among the study groups. Of note, adverse events were comparable between pigs receiving saline and AMK.

**Table 1:** Ventilator settings and adverse effects during nebulization

	Control	MEM	MEM + AMK	*p*-value
Group size, *n*	39	42	42	
Ventilator setting				
RR (breaths/min)	24.1 ± 4.4	26.6 ± 9.7	25.6 ± 6.2	0.28
Inspiratory flow (L/min)	30.3 ± 5.6	33.7 ± 11.8	31.1 ± 7.0	0.18
PEEP (cmH_2_O)	9.9 ± 1.1	8.7 ± 1.4*	9.1 ± 0.9^†^	< 0.001
Adverse effects, *n* (%)				
Coughing	2 (5.1)	1 (2.4)	1 (2.4)	0.73
Bronchoconstriction	0.0	0.0	0.0	1.00
Oxygen desaturation	0.0	0.0	2 (4.8)	0.14
Increased mucus production	26 (66.7)	32 (76.2)	31 (73.8)	0.61

Data are reported as means and standard deviations, medians (interquartile range), or (for adverse effects) the incidence among all nebulizations per group

AMK, inhaled amikacin; MEM, IV meropenem; PEEP, positive end-expiratory pressure; RR, respiratory rate

Post hoc analysis with Bonferroni correction: *p < 0.05 control versus MEM

^†^*p* < 0.05 control versus MEM + AMK

### Primary outcome

#### Lung *P*. *aeruginosa* burden

**Fig. 1:**
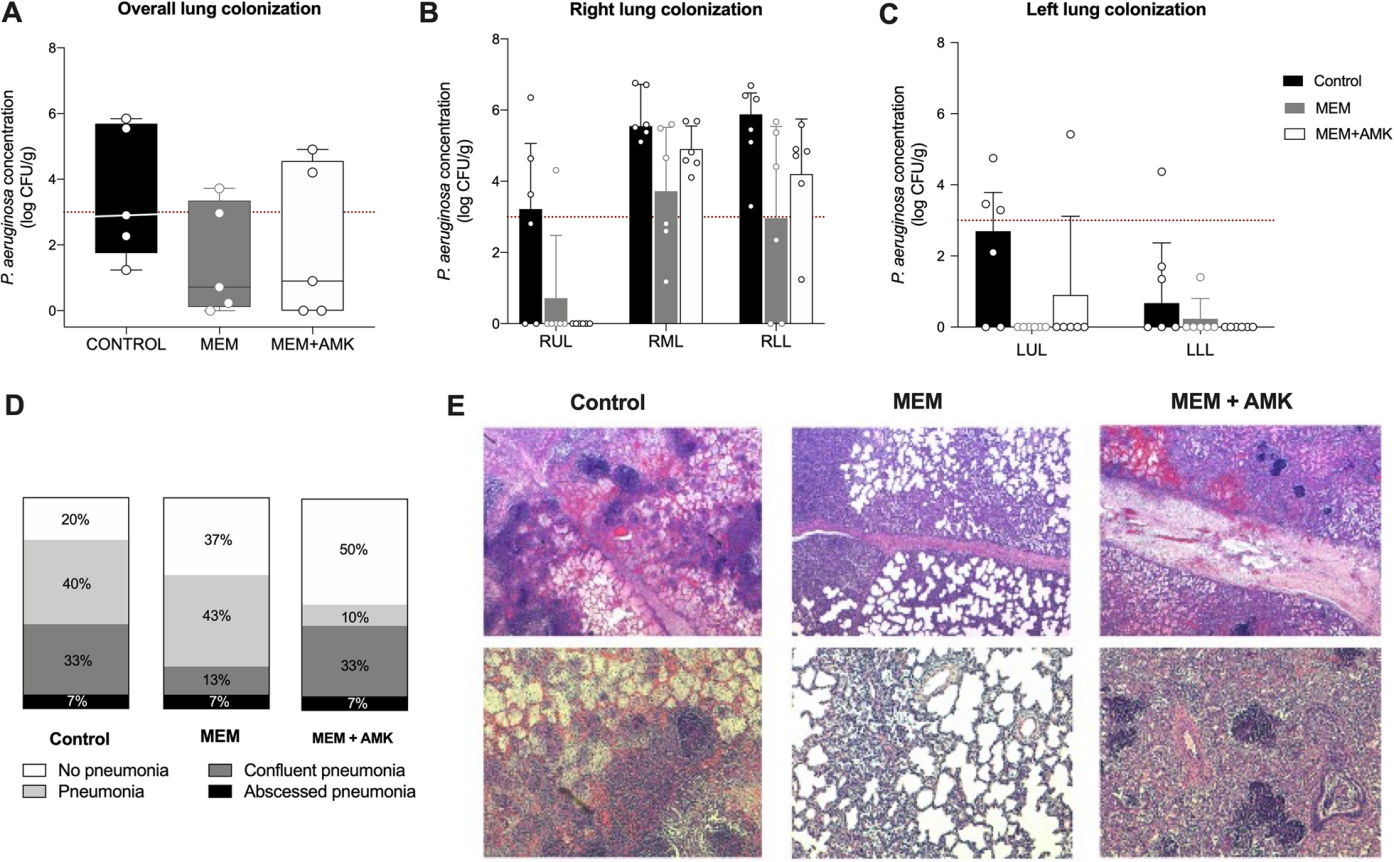
Lung tissue *P. aeruginosa* burden. **A**–**C** Graphs depict overall, right, and left lung tissue *P. aeruginosa* colonization, respectively. The red dashed line represents the diagnostic clinical cut-off value for infection. In each group, the median value is indicated by the center horizontal line, and the 95% confidence intervals are indicated by the lower and upper horizontal lines. Red dots represent new isolates resistant to meropenem. **D** Histopathological analysis of the 90 tissue samples. **E** Representative photomicrographs of pulmonary tissue retrieved at autopsy. The lung injury score is reported per study group: the CONTROL and MEM + AMK groups show a pattern of confluent pneumonia with bacteria and inflammatory cells between interlobular septa; by contrast, the MEM group shows A more predominant histopathology pattern of pneumonia with inflammatory cells within the alveolar regions, but not confluent through the interlobular septal region. Hematoxylin & eosin staining. Magnification: Upper section =  × 40; Lower section =  × 100. Abbreviations: CONTROL, control; MEM, IV meropenem group; MEM + AMK, meropenem and inhaled amikacin group

Figure 1 shows the differences in right lung tissue *P. aeruginosa* concentration among study groups (*p* = 0.025), with no statistically significant post hoc differences found between the MEM and MEM + AMK groups (*p* > 0. 99). Overall, 88.9%, 61.1%, and 66.7% of the right lung lobes were colonized by *P. aeruginosa* in the CONTROL, MEM, and MEM + AMK groups, respectively (*p* = 0.16). Likewise, the left lung (Fig. 1) differed significantly among study groups (*p* = 0.033), without significant variations between antimicrobial-treated animals: 50% in the CONTROL group were colonized by *P. aeruginosa* compared with 8.3% each in the remaining groups (*p* = 0.027). Overall, the median *P. aeruginosa* tissue concentration across all pulmonary lobes was 0.72 (0.12–3.35) in the MEM group, 0.90 (0–4.55) in the MEM + AMK group, and 2.91 (1.75–5.69) log_10_ CFU/g in the CONTROL group (*p* = 0.009).

An increase in the MIC of meropenem was found in three isolates, with two cases from the MEM group and one from the MEM + AMK group. Interestingly, left lung colonization was driven by MEM-resistant isolates in both treated groups.

Figure 1 also shows the results of the histopathological analysis of the 90 lung tissue samples. Significant differences in histological features were found among the therapeutic groups (*p* = 0.038). Confluent pneumonia with bacteria and inflammatory cells between interlobular septa was observed in the CONTROL and MEM + AMK groups, whereas the MEM group showed a more predominant histopathology pattern of pneumonia with inflammatory cells within the alveolar regions and not confluent through the interlobular septal region. The lung appearance, lung/body weight ratio, and signs of pneumonia at gross examination during autopsy are detailed in Additional file 1: Figure S2. Signs of pneumonia were less frequent in the MEM + AMK group.

### Secondary outcomes

#### Microbiological studies

**Fig. 2:**
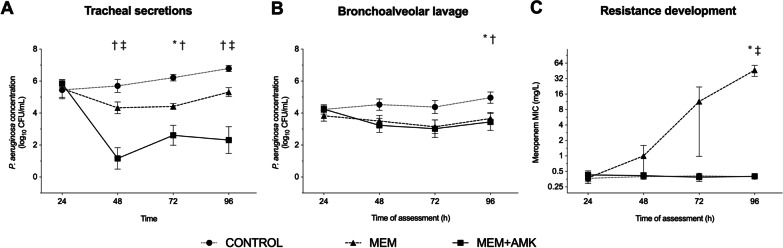
*P. aeruginosa* concentrations in tracheal secretions and bronchoalveolar lavage fluids*. P. aeruginosa* concentrations (log CFU/mL) and MICs are plotted as line graphs with means and standard errors of the mean. **A** *P. aeruginosa* concentrations in tracheal secretions differed among the study groups (*p* < 0.001) and over time (*p* < 0.001). Statistical significance against the CONTROL group for the MEM and MEM + AMK groups are shown by an asterisk and dagger, respectively. The double dagger shows a significant reduction of *P. aeruginosa* burden in the MEM + AMK group compared with the MEM group at 48 h (*p* < 0.001) and 96 h (p = 0.001). **B** Equally, *P. aeruginosa* concentrations in BAL fluids varied among treatments (*p* = 0.012), being significantly decreased in the MEM and MEM + AMK groups compared with the CONTROL group at 96 h. **C** The MIC of MEM for *P. aeruginosa* isolates did not change from that for the inoculated strain in the CONTROL and MEM + AMK groups, but increased to 11.5 ± 10.5 mg/L at 72 h and 46.8 ± 11.26 mg/L at 96 h in the MEM group. *p < 0.05 CONTROL versus MEM; ^†^*p* < 0.05 CONTROL versus MEM + AMK; ^‡^*p* < 0.05 MEM versus MEM + AMK. Abbreviations: AMK, inhaled amikacin; BAL, bronchoalveolar lavage; CFU, colony-forming unit; CONTROL, control; MEM, IV meropenem; MIC, minimum inhibitory concentration

Figure 2 shows the colonization of tracheal aspirates and bronchoalveolar lavage (BAL) fluids by *P. aeruginosa* colonization throughout the study. *P. aeruginosa* colonization within tracheal secretions differed among study groups (*p* < 0.001). Specifically, MEM + AMK therapy had bactericidal effects on tracheal secretions, contrasting with CONTROL (*p* < 0.001) and MEM (*p* = 0.002) throughout. The *P. aeruginosa* concentration in BAL fluids varied among the groups (*p* = 0.012). Compared with the CONTROL group, MEM alone (*p* = 0.027) and combined with AMK (*p* = 0.026) had antipseudomonal effects, though without differences between treated animals. Unexpectedly, *P. aeruginosa* was detected in only two animals in the MEM group and one in the MEM + AMK group (*p* = 0.77).

The MIC of MEM for *P. aeruginosa* isolates remained unchanged from the inoculated strain in the CONTROL and MEM + AMK groups, but increased significantly in the MEM group (*p* = 0.037). Specifically, the MIC in isolates from animals in the MEM group reached 64 (18–64) mg/L at the end of the study.

#### Antibiotic pharmacokinetics

**Fig. 3:**
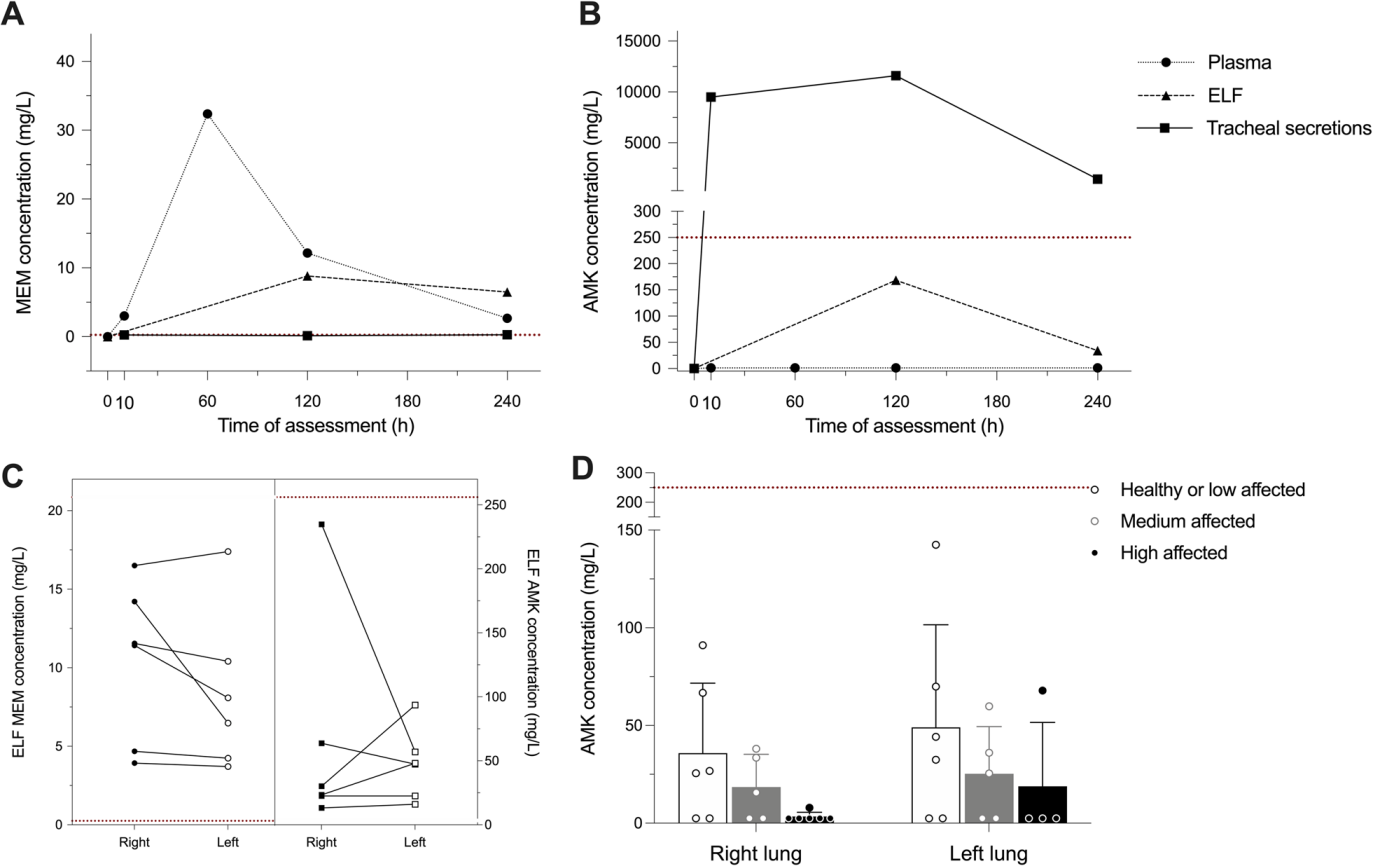
Antimicrobial pharmacokinetics. **A**, **B** AMK and MEM concentrations among study groups by assessment time and matrix. Of note, the AMK concentration only exceeded its MIC for *P. aeruginosa* in tracheal secretions. **C** ELF drug concentrations in the right medium (infected) and left upper (non-infected) lobes did not differ when analyzing paired concentrations (*p* = 0.63). **D** Non-significantly higher AMK tissue concentrations were observed in more preserved zones. Abbreviations: AMK, inhaled amikacin; ELF, epithelial lining fluid; MEM, IV meropenem; MIC, minimum inhibitory concentration

Figure 3 depicts the concentrations of AMK and MEM in plasma, ELF, and tracheal secretions. Of note, we used a *P. aeruginosa* strain with an MIC of 0.25 mg/L for MEM and administered MEM at a dose of 25 mg/Kg every 8 h, aiming to achieve a drug concentration greater than the MIC for at least 6 h [15]. The plasma MEM concentration was 2.67 ± 2.11 µg/mL 4 h after administration, while AMK was not detected at any time. The ELF concentrations of AMK and MEM were 41.2 ± 37.8 and 6.47 ± 4.96 µg/mL, respectively (*p* = 0.002). No paired differences in BAL concentration were found between infected and non-infected lungs (*p* = 0.63). MEM was not detected in 4 of 6 tracheal aspirates 4 h after dose, while two samples had low MEM levels. By contrast, the median (IQR; min–max) AMK concentrations in tracheal aspirates were 4310 (12.5–26,900; 12.5–33,800) mg/L.

Finally, MEM was detected in none of the pulmonary tissue samples of any lung or in the most or least affected areas, while AMK was detected in some tissue samples (Fig. 3). Although not significant (*p* = 0.088), more preserved areas showed higher concentrations of AMK than damaged regions.

#### Clinical variables

Table 2 summarizes the clinical parameter data. Study interventions reduced only the quantity of tracheal secretions (*p* < 0.001), while anemia was more pronounced in the MEM + AMK group (*p* < 0.001). Finally, creatinine levels were higher in the MEM and MEM + AMK groups than in the CONTROL group (*p* = 0.015), but without significant differences between the treatment groups.

**Table 2:** Clinical parameters

	Control	MEM	MEM + AMK	*p*-value
Temperature (°C)	38.3 ± 0.7	38.3 ± 0.6	38.1 ± 0.6	0.06
Tracheal secretions				
Quantity	2 (1–3)	1 (0–1)*	1 (0–1)^†^	< 0.001
Purulent (%)	100	98.4	94.6	0.11
WBC (10^9^/L)	13.3 (8.6–30.2)	16.0 (10.4–24.0)	13.6 (7.5–23.7)	0.91
Hemoglobin (g/L)	9.2 ± 0.9	9.4 ± 1.1	7.9 ± 1.4^†, ‡^	< 0.001
Platelets (10^9^/L)	313.3 ± 125.6	272.0 ± 104.0	249.4 ± 142.2	0.07
Creatinine (mg/dL)	1.00 (0.90–1.09)	1.07 (0.94–1.25)*	1.07 (1.01–1.22)^†^	0.017
PT (sec)	11.4 (10.5–12.0)	11.8 (10.5–13.8)	11.3 (10.5–12.4)	0.28

Data are reported as means ± standard deviations or as medians (interquartile range) for normally and non-normally distributed parameters, respectively. The PT reference range is 9–12 s in pigs

AMK, inhaled amikacin; MEM, IV meropenem; PT, prothrombin time; PTT, thromboplastin time

Post hoc analysis with Bonferroni correction: *p < 0.05 control versus MEM

^†^*p* < 0.05 control versus MEM + AMK

^‡^*p* < 0.05 MEM versus MEM + AMK

#### Pulmonary mechanics and hemodynamics

Additional file 1: Figure S3 shows the data for pulmonary function and mechanics. This highlights that oxygenation improved in the MEM and MEM + AMK groups, but without inhaled AMK offering additional benefit. We observed a comparable increase in the pulmonary shunt for the CONTROL group (*p* = 0.007). As shown in Additional file 1: Table S2, hemodynamic parameters did not improve in the MERO + AMK group, especially in comparison with the MEM group.

#### Inflammatory markers

**Fig. 4:**
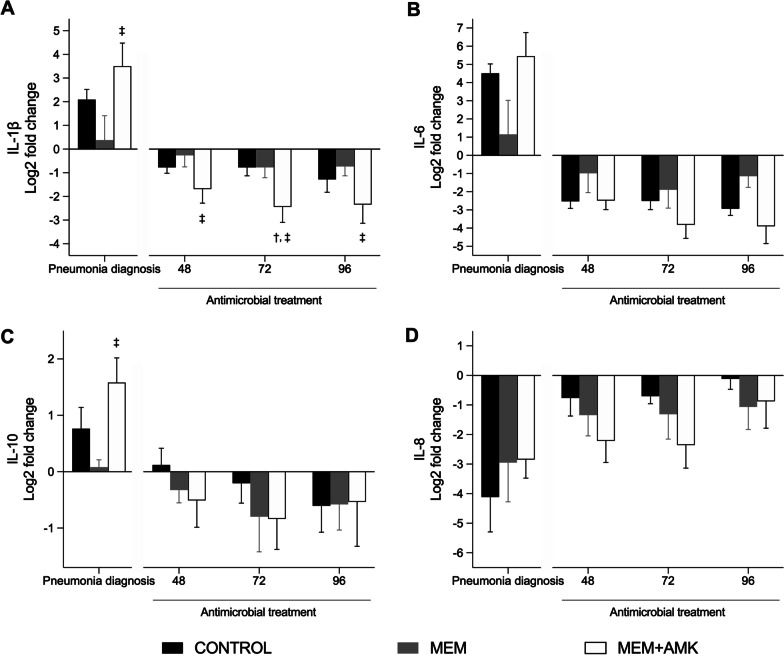
Inflammatory markers. Bars show the fold-change from baseline (log2) and pneumonia diagnosis among study groups at the diagnosis of pneumonia and treatment with antimicrobials timepoints. **A** IL-1β varied significantly among study groups (*p* = 0.025) and over time (*p* = 0.043). Post hoc comparisons confirmed that IL-1β was downregulated by MEM + AMK treatment at 48, 72, and 96 h compared with the CONTROL and MEM groups. **B**–**D** IL-6, IL-8, and IL-10 did not vary among study groups, but did show a downward trend over time. Post hoc analysis with Bonferroni correction: *p < 0.05 CONTROL versus MEM; ^†^*p* < 0.05 CONTROL versus MEM + AMK; ^‡^*p* < 0.05 MEM versus MEM + AMK. Abbreviations: AMK, inhaled amikacin; CONTROL, control; IL, interleukin; MEM, IV meropenem

Figure 4 shows the serum concentrations of systemic inflammatory markers. The *P. aeruginosa* challenge caused a significant increase in all assessed cytokines, except interleukin (IL) 8. However, antibiotic treatments decreased serum levels of IL-1β, IL-6, and IL-10. Of note, systemic IL-1β was significantly downregulated by inhaled AMK compared with the control and MEM groups (*p* = 0.025). Similarly, systemic IL-6 and Il-10 were upregulated at the diagnosis of pneumonia and showed downward trends throughout treatment, but without showing significant differences among study groups.

## Discussion

This study describes the development of a porcine model of monolateral severe *P. aeruginosa* pneumonia resistant to AMK and susceptible to MEM, which we used to explore the inhaled amikacin therapy. Our findings demonstrate that MEM treatment drove a reduction in lung tissue concentration of *P. aeruginosa*, and that adding inhaled AMK as adjunctive therapy only reduced the bacterial burden of tracheal secretions. Unfortunately, we found no effect of the inhaled therapy on preventing dissemination compared with systemic monotherapy, although histological analysis revealed significantly fewer signs of pneumonia than in the CONTROL group.

Our results are consistent with the latest data from randomized controlled trials [14, 19]. Indeed, in the IASIS trial [14] found only a marginal effect with inhaled adjunctive amikacin/fosfomycin and only in tracheal aspirate samples, of which significantly fewer were positive on days 3 and 7 compared to placebo. In the INHALE trial [19], which used the same dosage of AMK (i.e., 400 mg every 12 h), did show more frequent eradication among patients infected with *P. aeruginosa* and treated with inhaled AMK; however, this did not translate to improved survival. The reason why inhaled antibiotics do not prove benefits may be related to the susceptibility of pathogens [25]. All enrolled patients in both trials were infected by pathogens susceptible to the intravenous antibiotics, as *P. aeruginosa* was susceptible to meropenem in our model. Furthermore, *P. aeruginosa* was resistant to AMK. Therefore, inhaled adjunctive therapy, even if effective, was unlikely to have a measurable effect. In a recent meta-analysis of six randomized controlled trials, inhaled adjunctive therapy achieved higher clinical resolution (odds ratio, 1.96; 95% CI 1.30–2.96) in patients with pneumonia due to MDR pathogens, albeit not in those with susceptible bacteria [26].

Even with 72 h of IV MEM therapy, inhaled AMK suppressed the emergence of the MEM-resistant subpopulation compared with systemic MEM therapy alone. The most recent clinical studies indicated that inhaled treatment may hinder the development of resistance [27]. In our study, only one animal in the AMK group developed MEM resistance, and of note, this was in the only animal in which *P. aeruginosa* colonized the left non-infected lung, suggesting a role for acquired resistance.

As expected, tracheal secretions revealed high AMK concentrations and MEM concentrations below the limit of detection (0.10 mg/L). AMK was not detected in plasma though out the experiment, suggesting poor AMK translocation from the lungs into the bloodstream, reinforcing the idea that using such drugs can prevent systemic toxicity [18]. In contrast, high AMK concentrations in tracheal secretions correlate with rapidly sterilized bronchial secretions. These results should not be neglected as they suggest a favorable prophylactic effect on the progression from ventilator-associated tracheobronchitis (VAT). Indeed, Palmer et al*.* showed a faster resolution of signs of infection when assessing the effects of adjunctive nebulized antibiotic therapy in patients with VAT [28].

AMK concentrations in the ELF were significantly higher than MEM concentrations, but we still below the MIC. The large difference between the MICs of MEM and AMK (i.e., 0.5 vs 256 mg/L, respectively) means that although the AMK concentration in the ELF reached higher figures, the maximum concentration (at least 10 times the MIC of the infecting pathogen) was not achieved. By contrast, the MEM free fraction concentration remained above the MIC achieved 100% of the time in the ELF.

We also measured the AMK and MEM levels in both the infected and non-infected lungs, and found similar concentrations in the ELF bilaterally. However, when we measured the AMK concentrations in tissue samples, we found a non-significant trend of higher antimicrobial concentration in the more preserved areas. This indicated that inhaled AMK did not efficiently reach poorly aerated lung parenchyma. The deposition of inhaled drugs in the lungs and in areas of pneumonia with loss of aeration is often questionable and may constitute a major limitation when using this approach to treat ventilated patients [25]. Indeed, previous studies have shown that inhaled antibiotics may not reach consolidated lung segments [12, 15], with research by Elman et al. in infected piglets revealing that the pulmonary concentration of inhaled AMK was reduced in cases with extensive parenchymal infection [12]. In our setting, probably the infected lung has different lung characteristics with a lower compliance and higher airway resistance reducing drug distribution [29].

Notably, we did not change the ventilator settings during nebulization, including the humidification and ventilator circuit, according to the manufacturer’s instructions. The European Investigators Network for Nebulized Antibiotics in Ventilator-associated Pneumonia agreed on specific recommendations for ventilator settings [30], but we did not follow all of these in our protocol. Specifically, the recommendations specify that the mesh nebulizer should be placed 10–15 cm before the Y-piece on the inspiratory limb, whereas we placed it only a few centimeters from the Y-piece [31]. Also, turning off active humidification is recommended during nebulization to avoid hygroscopic growth and a rainout effect in the circuits and the endotracheal tube [32]. Although the penetration of AMK may be modified by using our ventilator configuration, the impact of this was not investigated.

As for how the study treatments affected other clinical, pulmonary mechanics, and hemodynamics variables, we found that even the most efficacious treatment had minimal impact. Only oxygenation was improved in treated animals, but without any additional benefit associated with the use of inhaled AMK, though it did have a slight effect on the systemic IL-1β downregulation.

Finally, no adverse effects were reported. Creatinine levels were higher in both the MEM and MEM + AMK groups than in the CONTROL group, but without significant differences between the treatment groups, or indeed, evidence of nephrotoxicity [17, 18].

Our findings help to delineate where inhaled antibiotics might have utility in the management of VAP or ventilated HAP, whereas they may be limited to patients with difficult-to-treat pathogens. Although their impact on preventing dissemination seems to be trifling, the high efficacy on tracheal secretions may provide a therapeutic opportunity for VAT. Second, as observed over the 100 h of this study, the use of a nebulized antibiotic may impede resistance to IV antibiotics in selected high-risk patients or in intensive care units with high MDR rates [33]. Third, future studies should explore the optimal method for measuring inhaled antibiotic concentrations to ensure adequate dosage regimens to reach distal portions of highly infected pulmonary regions [34].

This study presents some limitations that deserve further discussion. First, we used a 72-h course of therapy, which is unlikely in the most probable clinical scenario. Second, the susceptibility of *P. aeruginosa* to MEM makes it difficult to show the window of efficacy for inhaled AMK. Furthermore, only one strain was tested. Third, pharmacokinetic analyses were performed for only 4 h after dose administration, limiting the picture of antimicrobial distribution to a brief period. Moreover, the differences between ELF and tissue samples may suggest that the former are not the best surrogate of pulmonary concentrations, particularly for inhaled drugs with a high risk of bronchial tree contamination and where their deposition is important [34, 35]. The AMK dose may also be questioned because it is a concentration-dependent antibiotic, and twice-daily administration similar to the IASIS trial may have produced variable ELF concentrations [14, 19]. Finally, we studied young animals with no comorbidities under deep sedation. Moreover, anatomical differences of the tracheobronchial tree of piglets from human anatomy are critical factors that may affect lung deposition of inhaled particles [36].

## Conclusions

In a swine model of monolateral MDR *P. aeruginosa* pneumonia resistant to the inhaled AMK and susceptible to the IV antibiotic, the use of AMK as an adjuvant treatment offered no benefits in terms of pulmonary tissue colonization or the prevention of pathogen dissemination. However, it improved bacterial eradication in the proximal airways and hindered antibiotic resistance and showed a non-significant trend to producing higher AMK concentrations in more preserved lung areas.

## Supplementary Information

**Additional file 1.** Supplementary methods and results

## Data Availability

The datasets used and/or analyzed during the current study are available from the corresponding author on reasonable request.

## Abbreviations

ALAT: Asociacion Latinoamericana del Torax
AMK: Amikacin
BAL: Bronchoalveolar lavage
CFU: Colony-forming units
CRE: Carbapenem-Resistant Enterobacterales
ELF: Epithelial lining fluid
ERS: European Respiratory Society
ESCMID: European Society of Clinical Microbiology and Infectious Diseases
ESICM: European Society of Intensive Care Medicine
FiO_2_: Fraction of oxygen in the inhaled gas
HAP: Hospital-acquired pneumonia
IL: Interleukin
IQR: Interquartile range
IV: Intravenous
MDR: Multidrug resistant
MEM: Meropenem
MIC: Minimum inhibitory concentration
PaO_2_: Arterial partial pressure of oxygen
PEEP: Positive end-expiratory pressure
RR: Respiratory rate
SEM: Standard errors of the mean
VAP: Ventilator-associated pneumonia
VAT: Ventilator-associated tracheobronchitis
