# Supplementary material for: Inhaled amikacin for pneumonia treatment and dissemination prevention: an experimental model of severe monolateral Pseudomonas aeruginosa pneumonia
Source: Crit Care. 2023 Feb 14;27:60. doi: 10.1186/s13054-023-04331-x (PMC9930251; doi:10.1186/s13054-023-04331-x)
Supplement: Supplementary file 1 — Additional file 1. Supplementary methods and results [file 13054_2023_4331_MOESM1_ESM.docx]

Version date 28/12/2022

Submission: Critical Care

**Inhaled Amikacin for Pneumonia Treatment and Dissemination Prevention: An Experimental Model of Severe Monolateral *Pseudomonas aeruginosa* Pneumonia**

Ana Motos^1-4*^, Hua Yang^1-3*^, Gianluigi Li Bassi^5,14^, Minlan Yang^1-3^, Andrea Meli^1,6^, Denise Battaglini^1,3,7^, Roberto Cabrera^1,2,4^, Joaquim Bobi^1,2^, Francesco Pagliara^1,7^, Gerard Frigola^8^, Marta Camprubí-Rimblas^4,9^, Laia Fernandez-Barat^1-4^, Montserrat Rigol^1,2^, Antoni Ferrer-Segarra^1,10^, Kasra Kiarostami^1-3^, Daniel Martinez^8^, David P Nicolau^11^, Antonio Artigas^4,9^, Paolo Pelosi^7,12^, Jordi Vila^13^, Antoni Torres^1-4^

*AM and HY equally contributed

^1^ Hospital Clínic, Thorax Institute, Pneumology Department, Barcelona, Spain.

^2^ Institut d'Investigacions Biomèdiques August Pi i Sunyer (IDIBAPS), Barcelona, Spain.

^3^University of Barcelona, Barcelona, Spain.

^4^Centro de Investigación Biomedica En Red- Enfermedades Respiratorias (CIBERES), Barcelona, Spain.

^5^ BITRECS Fellow, Institut d'Investigacions Biomèdiques August Pi i Sunyer (IDIBAPS), Barcelona, Spain

^6^ Fondazione IRCCS Cà Granda Ospedale Maggiore Policlinico Internal Medicine Department, Respiratory Unit and Adult Cystic Fibrosis Center, and Department of Pathophysiology and Transplantation, Università degli Studi di Milano, Milan (Italy)

^7^ Anesthesia and Intensive Care, San Martino Policlinico Hospital, IRCCS for Oncology and Neurosciences, Genoa, Italy

^8^ Department of Pathology, Hospital Clinic, Barcelona, Spain

^9^ Critical Care Center, ParcTaulí Hospital Universitari, Institut d'Investigació i Innovació Parc Taulí (I3PT), Universitat Autònoma de Barcelona, Sabadell, Spain.

^10^ Anestesiologia i Reanimació. Hospital del Mar - Parc de Salut Mar, Barcelona, Spain

^11^ Center for Anti-Infective Research & Development, Hartford Hospital, Hartford, CT USA

^12^ Department of Surgical Sciences and Integrated Diagnostics (DISC), University of Genoa, Genoa, Italy

^13^ ISGlobal, Hospital Clínic-Universitat de Barcelona, Barcelona, Spain; Department of Clinical Microbiology, Centre for Biomedical Diagnosis, Hospital Clínic, Barcelona, Spain.

^14^ Critical Care Research Group, The Prince Charles Hospital, University of Queensland, Queensland University of Technology, UnitingCare Hospitals, Wesley Medical Research, Brisbane, Australia

**Running Head:** Inhaled Amikacin for pneumonia treatment.

Corresponding author: Antoni Torres, MD, PhD

Servei de Pneumologia i Al•lèrgia Respiratòria

Hospital Clínic

Calle Villarroel 170, Esc 6/8 Planta 2

08036 Barcelona (S*P. aeruginosa*IN)

Voice/Fax: 0034 932275549

Email: [atorres@clinic.cat](mailto:atorres@clinic.cat)

**Table S1.** **Comparison among groups upon diagnosis of pneumonia.**

|  | CONTROL | MEM | MEM+AMK | p-value |
| --- | --- | --- | --- | --- |
| Body Temperature (ºC) | 38.2[0.4] | 38.4[1.1] | 39.2[0.4] | 0.012 |
| Tracheal Secretions Quantity | 1.0[0.0] | 1.0[1.0] | 1.0[0.0] | 0.769 |
| Purulent Tracheal Secretions (%) | 100 | 94.8 | 89.6 | 0.471 |
| WBC (10^9^/L) | 11.6[29.9] | 18.7[12.9] | 7.5[1.4] | 0.416 |
| PaO_2_/FiO_2_ | 307.0 [36.2] | 303.6 [21.3] | 319.3 [112.8] | 0.843 |
| Lung Elastance (cm H_2_0/L) | 28.9[8.5] | 30.1[4.9] | 25.5[9.4] | 0.070 |

Table S1 caption: Data are reported as median [interquartile range]. WBC, white blood cells, PaO2/FiO2, ratio between arterial partial pressure of oxygen and inspiratory fraction of oxygen; MEM, intravenous meropenem; AMK+MEM, aerosolized amikacin and intravenous meropenem.

**Table S2. Hemodynamic parameters**

|  | CONTROL | MEM | MEM+AMK | P-value |
| --- | --- | --- | --- | --- |
| HR (beats/min) | 61.0± 9.1 | 66.0± 7.9 | 65.1± 13.5 | 0.401 |
| MAP (mmHg) | 72.2± 4.3 | 74.4± 7.3 | 72.2± 4.7 | 0.322 |
| MPAP (mmHg) | 20.5± 1.6 | 22.0± 0.8 | 20.7± 1.4 | 0.177 |
| CVP (mmHg) | 7.3± 0.8^ | 8.3± 0.4 | 8.5± 0.6 | 0.033 |
| PCWP (mmHg) | 10.6± 0.6 | 12.0± 0.3**†** | 10.6± 0.6 | 0.009 |
| CO (L/min) | 3.2± 0.3 | 3.1± 0.3 | 3.3± 0.7 | 0.722 |
| SVR (dynes/sec/cm^-5^) | 1713± 199 | 1871± 271 | 1626± 303 | 0.087 |
| PVR (dynes/sec/cm^-5^) | 265± 31 | 278± 38 | 253± 46 | 0.434 |
| Vasopressor Dependency Index  (µg/Kg/min) | 0.07 [0.29] | 0.06 [0.36] | 0.07 [0.62] | 0.985 |

Table S2 caption: Dat reported as mean ± standard deviation or median [interquartile range] for normally or not normally distributed parameters. MEM, meropenem group; MEM+AMK, meropenem and inhaled amikacin group; HR, heart rate; MAP, mean arterial pressure; MPAP, mean pulmonary arterial pressure; CVP, central venous pressure; PCWP, pulmonary capillary wedge pressure; CO, cardiac output; SVR, systemic vascular resistance; PVR, pulmonary vascular resistance. Of note, normal cardiac output in pigs of 30-35 Kg ranges between 2.0-3.0 L/min, while systemic and pulmonary vascular resistance range 1600-2400 and 500-600 dynes/sec/cm, respectively.

**Figure S1: Main Study Sequential Assessments of all 96 hours.** *Nebulization will be carried out every 12 hours (green circles), Meropenem IV, will be administered every 8 hours (yellow circles). ^ Upon the first nebulization, and the first administration of meropenem, analysis of plasma antibiotic concentrations at pre-dose, 10 minutes, 1, 2, 4 hours post dosing will be carried out. Additionally, antibiotics will be also quantified in tracheal aspirate at pre-dose, 10 minutes, 2, and 4 hours post dosing and in BAL at pre-dose, 2 and 4 hours post dosing.

**
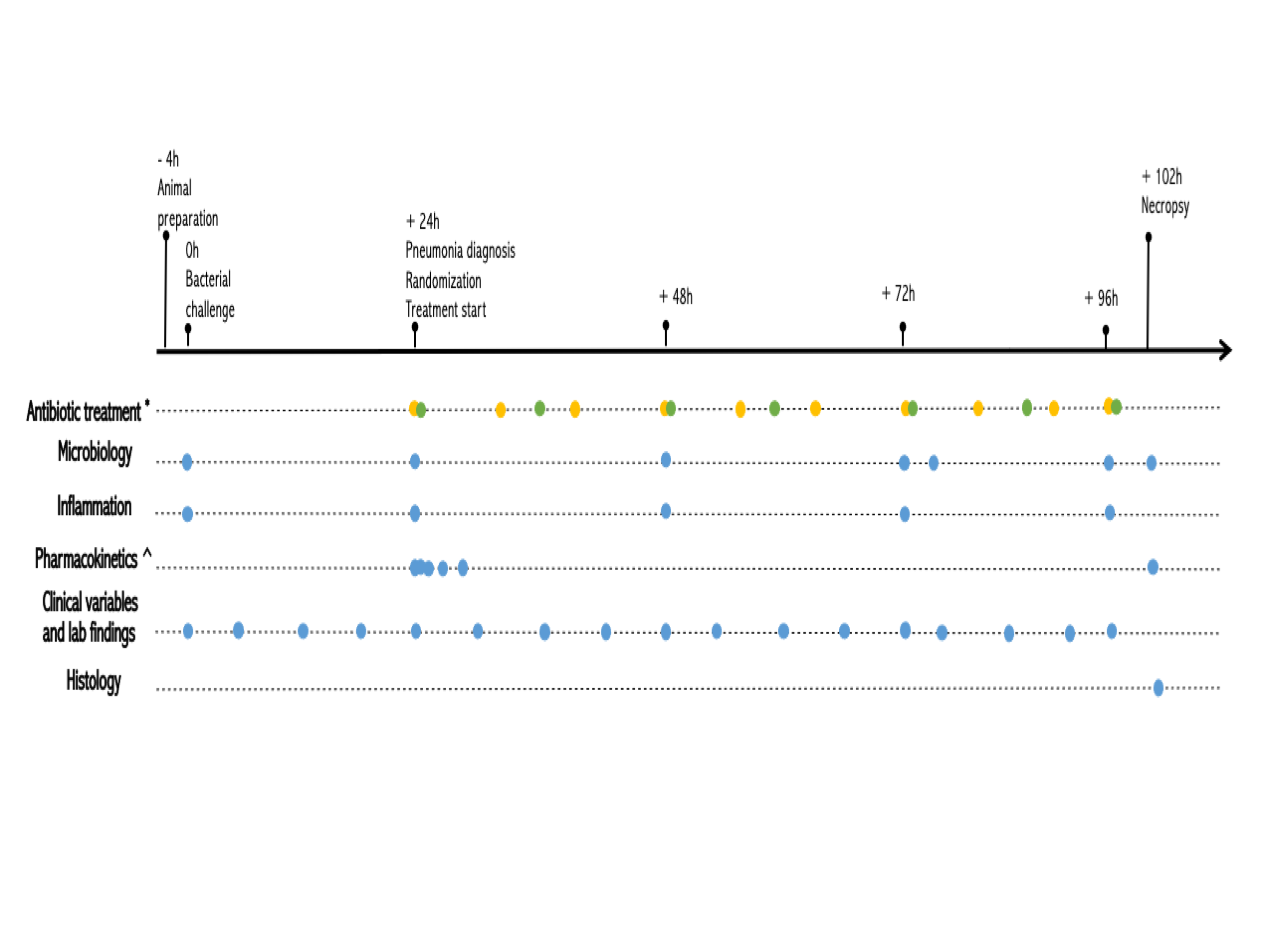
**

**Figure S2: Lungs appearance upon retrieval.** A)Weight lung/body ratio among study groups with significant differences (p=0.47). B) Macroscopic signs of pneumonia. Signs of pneumonia were less frequent in the MEM+AMK group (p=0.028). C) Pictures of lungs upon autopsy retrieval per study group. The model of monolateral pneumonia was developed through instillation of *Pseudomonas aeruginosa* into the right lung. MEM, meropenem group; MEM+AMK, meropenem and inhaled amikacin group.


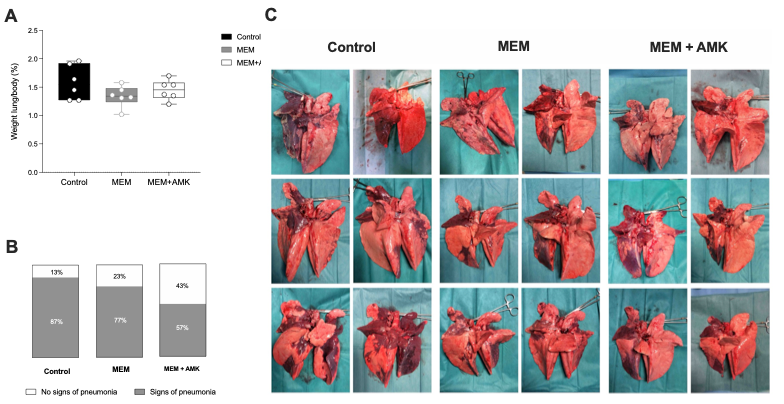


**Figure S3 Pulmonary function and mechanics.** Data reports mean values per each time point among study groups. A, Ratio of partial pressure of oxygen per inspiratory fraction of oxygen differed among study groups (p=0.007). * post-hoc comparisons with Bonferroni corrections, p<0.05 vs MEM+AMK. B, Pulmonary shunt was different among study groups (p=0.007). In particular, CONTROL group showed the highest figures. † post-hoc comparisons with Bonferroni corrections, p<0.05 vs MEM and MEM+AMK. C, Minute ventilation was not different among study groups (p=0.260). Similarly, D, lung elastance (p=0.426) did not differ among groups. Finally, E, inspiratory airflow resistance (p=0.079) and tissue resistance (p=0.967) were similar among study groups.
